# Distinctive personality patterns in patients with chronic viral hepatitis

**DOI:** 10.1186/1471-2334-14-S7-O13

**Published:** 2014-10-15

**Authors:** Ioana-Catrinel Cercel, Șerban Polli, Oana Săndulescu, Anca Streinu-Cercel, Ielița Jivcovici, Daniela Manolache, Adrian Streinu-Cercel

**Affiliations:** 1 National Institute for Infectious Diseases "Prof. Dr. Matei Balş", Bucharest, Romania; 2 Carol Davila University of Medicine and Pharmacy, Bucharest, Romania

## Background

Minnesota Multiphasic Personality Inventory (MMPI) is a tool that provides information on adult personality and psychopathology. We have performed a study to determine personality patterns in patients with chronic viral hepatitis.

## Methods

We assessed patients with chronic hepatitis B and C as well as a control group, using the MMPI-2 standardized psychometric tool. The subjects were evaluated during an appointment with the clinical psychologist in July 2014. For statistical purposes, the T score was used to compare the results between different groups.

## Results

We have evaluated 29 patients with chronic viral hepatitis (21 females and 8 males – 9 with HBV and 20 with HCV) and 28 subjects for the control group (18 females and 10 males). The mean age in patients with chronic hepatitis was 47.97±13.63 vs. 31.36±8.28 in the control group.

Comparing the results between the control group and the two subgroups with HBV and HCV infection we obtained the following statistically significant differences:

• The HBV group presented 31 scales with significant differences compared to controls, with elevated scores for: OBS (obsessiveness), DEP (depression), ANG (anger), TPA (type A personality) and TRT (negative treatment indicators) scales and subscales TRT1 (low motivation) and TRT2 (inability to disclose), etc.

• The HCV group presented 7 scales with significant differences compared to controls, with elevated scores mostly for scales related to the somatic connection.

We also identified statistically significant differences between the two chronic hepatitis subgroups (namely 27 scales related to OBS (obsessions), DEP1 (lack of drive), ANG2 (irritability), and CYN (cynicism).

## Conclusion

Based on the results of this study, we can conclude that there seem to be certain distinctive personality patterns in patients with chronic HBV and HCV infection. These results outline the utility of psychological assessment with specialized instruments in order to include psychological variables in the clinical framework and to improve the clinical decision-making process.

